# Ouabaïne

**Published:** 1891-01-17

**Authors:** 


### THERAPEUTICS.

### OUABAÏNE.

Ouabaïne is an alkaloid obtained from an African shrub. It is the active principle of an arrow poison used by the Somali natives, South-Eastern Africa. It exists in the form of rectangular crystals, excessively thin, white, odourless, and transparent, and is an extremely deadly poison, a milligramme or two, introduced into the blood by a scratch or otherwise, causes death.

This alkaloid was first prepared and described by M. Arnaud. It is insoluble in ether and chloroform, and is only very slightly soluble in absolute alcohol. Its best solvent is partly concentrated alcohol. Its chemical formula is approximately $C_{30}\ H_{46}\ (O\ H_2)_7$.

Its physiological action has been investigated by Professor Guy, of Paris, and in its properties it has been found to closely resemble strophanthus, the ouabaio shrub belonging to ths same botanical order as strophanthus.

In frogs the heart is arrested in eight or nine minutes by the subcutaneous injections of $\frac{1}{5500}$ of a grain, and in about six minutes by the injection of about $\frac{1}{2250}$ of a grain, only half the dose of strophanthus required to produce similar effects.

It acts by gradually causing paralysis of the ventricles, the auricles continuing in action for some time after the arrest of ventricular action. The lungs become pale and anæmic.

In rabbits similar effects were obtained, when $\frac{1}{700}$ of a grain per two lb. of the weight of the animal was used. The principal effect of $\frac{1}{10}$ of a grain injected in a dog was marked by slowing of the respirations without much cardiac disturbance, lasting for a few minutes and then ceasing. Larger doses first quickened the respirations, and then caused them to become gradually slower until they ceased, the heart's pulsations continuing for a few minutes after their arrest. In a case when artificial respiration was kept up, the heart's action did not cease, and in a few minutes the respirations were maintained as well as the heart's beats.

The poison acts much less powerfully when introduced by the stomach; thus a dog weighing 3½ lbs. exhibited all the symptoms of ouabaïne poisoning, but survived after being given about $\frac{1}{10}$ grain of the alkaloid.

From its powerful action on the respiratory centres ouabaïne has been suggested as a remedy likely to prove useful in asthma and pertussis, and in cardiac diseases, as a substitute for digitaline and strophanthine. It has been clinically investigated by Drs. Watson Williams, of Bristol, and Gemmell, of Glasgow, and others.

In some cases of spasmodic asthma the remedy gives excellent results, especially when administered during an attack. The extremely poisonous properties of this drug must be borne in mind when prescribing it. For this reason only the active principle should be used, as in the standardised liquor ouabaïne, prepared by Messrs. Christy and Co., each drachm of which contains $\frac{1}{5000}$ grain. One or two drachms of liquor ouabaïne may be given every quarter of an hour till the attack passes off, not more than three doses being allowed at any one time. Generally a single dose is sufficient to give marked relief.

It may be given regularly in the intervals between the attacks, to prevent their recurrence, but it is often disappointing as a prophylactic in asthma. We know of no remedy which has such a rapid, beneficial action in pertussis in all the stages. In some of our cases onabaïne seemed to abort the disease, and all, without exception, were remarkably improved, the paroxysms becoming less frequent and much milder. Dr. Gemmell has treated forty-nine cases. He has adopted $\frac{1}{1000}$ grain every three hours as a proper dose for a child of five years.

As a cardiac remedy, it is well worth careful investigation, although its sphere is limited. It relieves functional palpitation, especially palpitation due to the irritation of the vagus endings at the cardiac end of the stomach. It is much inferior to digitalis, strophanthus, barium chloride, convallaria, etc., in mitral regurgitation. In some cases relief was obtained and deficient secretion of urine partially overcome, but with our very much more efficient drugs at hand, onabaïne cannot be recommended for such conditions. It is rather in aortic valve disease, associated with atheromatous arteries, that ouabaïne is indicated. It is a pure heart tonic, and does not appear to raise the arterial tension generally. For cardiac asthma it may be used in combination with other remedies, such as trinitrin. It is generally inferior to nitrite of amyl and trinitrin in angina, though in some cases it will be found to give relief in half a-minute, and, given regularly, will tend to keep off the slighter degrees of angina so frequently associated with organic heart disease, without culminating in acute paroxysms.

